# High-dimensional comparison of monocytes and T cells in post-COVID and idiopathic pulmonary fibrosis

**DOI:** 10.3389/fimmu.2023.1308594

**Published:** 2024-01-16

**Authors:** Grace C. Bingham, Lyndsey M. Muehling, Chaofan Li, Yong Huang, Shwu-Fan Ma, Daniel Abebayehu, Imre Noth, Jie Sun, Judith A. Woodfolk, Thomas H. Barker, Catherine A. Bonham

**Affiliations:** ^1^Department of Biomedical Engineering, University of Virginia, Charlottesville, VA, United States; ^2^Division of Asthma, Allergy and Immunology, Department of Medicine, University of Virginia, Charlottesville, VA, United States; ^3^Carter Immunology Center, University of Virginia, Charlottesville, VA, United States; ^4^Division of Infectious Disease and International Health, Department of Medicine, University of Virginia, Charlottesville, VA, United States; ^5^Division of Pulmonary and Critical Care Medicine, University of Virginia, Charlottesville, VA, United States; ^6^Division of Pulmonary and Critical Medicine, Department of Medicine, Mayo Clinic, Rochester, MN, United States

**Keywords:** COVID – 19, pulmonary fibrosis, monocytes, idiopathic pulmonary fibrosis, single cell RNA sequencing (scRNA), peripheral blood mononuclear cells

## Abstract

**Introduction:**

Up to 30% of hospitalized COVID-19 patients experience persistent sequelae, including pulmonary fibrosis (PF).

**Methods:**

We examined COVID-19 survivors with impaired lung function and imaging worrisome for developing PF and found within six months, symptoms, restriction and PF improved in some (Early-Resolving COVID-PF), but persisted in others (Late-Resolving COVID-PF). To evaluate immune mechanisms associated with recovery versus persistent PF, we performed single-cell RNA-sequencing and multiplex immunostaining on peripheral blood mononuclear cells from patients with Early- and Late-Resolving COVID-PF and compared them to age-matched controls without respiratory disease.

**Results and discussion:**

Our analysis showed circulating monocytes were significantly reduced in Late-Resolving COVID-PF patients compared to Early-Resolving COVID-PF and non-diseased controls. Monocyte abundance correlated with pulmonary function forced vital capacity and diffusion capacity. Differential expression analysis revealed MHC-II class molecules were upregulated on the CD8 T cells of Late-Resolving COVID-PF patients but downregulated in monocytes. To determine whether these immune signatures resembled other interstitial lung diseases, we analyzed samples from Idiopathic Pulmonary Fibrosis (IPF) patients. IPF patients had a similar marked decrease in monocyte HLA-DR protein expression compared to Late-Resolving COVID-PF patients. Our findings indicate decreased circulating monocytes are associated with decreased lung function and uniquely distinguish Late-Resolving COVID-PF from Early-Resolving COVID-PF, IPF, and non-diseased controls.

## Introduction

Over 770 million cases of COVID-19 have been documented worldwide, and up to 30% of patients experience persistent symptoms months after illness (1–4). Pulmonary fibrosis is common in COVID-19 patients following hospitalization in the intensive care unit (ICU). Recent studies report 27% of computed tomography (CT)-scanned patients develop fibrosis during hospitalization, which increases to 33% six months after illness (5, 6). It is unknown what mechanisms govern resolution or persistence of pulmonary fibrosis associated with severe COVID-19 and whether COVID-associated pulmonary fibrosis (COVID-PF) is similar to progressive pulmonary fibrosis (7).

Recent evidence indicates that abnormal immune function plays a significant role in COVID-19 severity (8–11). For example, SARS-CoV-2 causes a robust inflammatory response within the lung that can lead to acute respiratory distress syndrome (ARDS), tissue damage, and long-term respiratory dysfunction (12–15). A growing number of studies have begun to characterize COVID-PF (16–21), but a comprehensive analysis of multiple cell types that distinguish poor outcomes within COVID-PF is critically needed to inform treatment.

Peripheral immune cells are an ideal tool for examining differences in the immune response as post-COVID-19 sequelae often manifests as a systemic disorder. Furthermore, profiling peripheral blood mononuclear cells (PBMCs) has yielded insights into immune dysregulation and markers that predict disease outcomes in patients with idiopathic pulmonary fibrosis (IPF), the most common fibrotic lung disease (22–26), setting a precedent that similar methodologies could be applied to COVID-PF.

**Figure 1:**
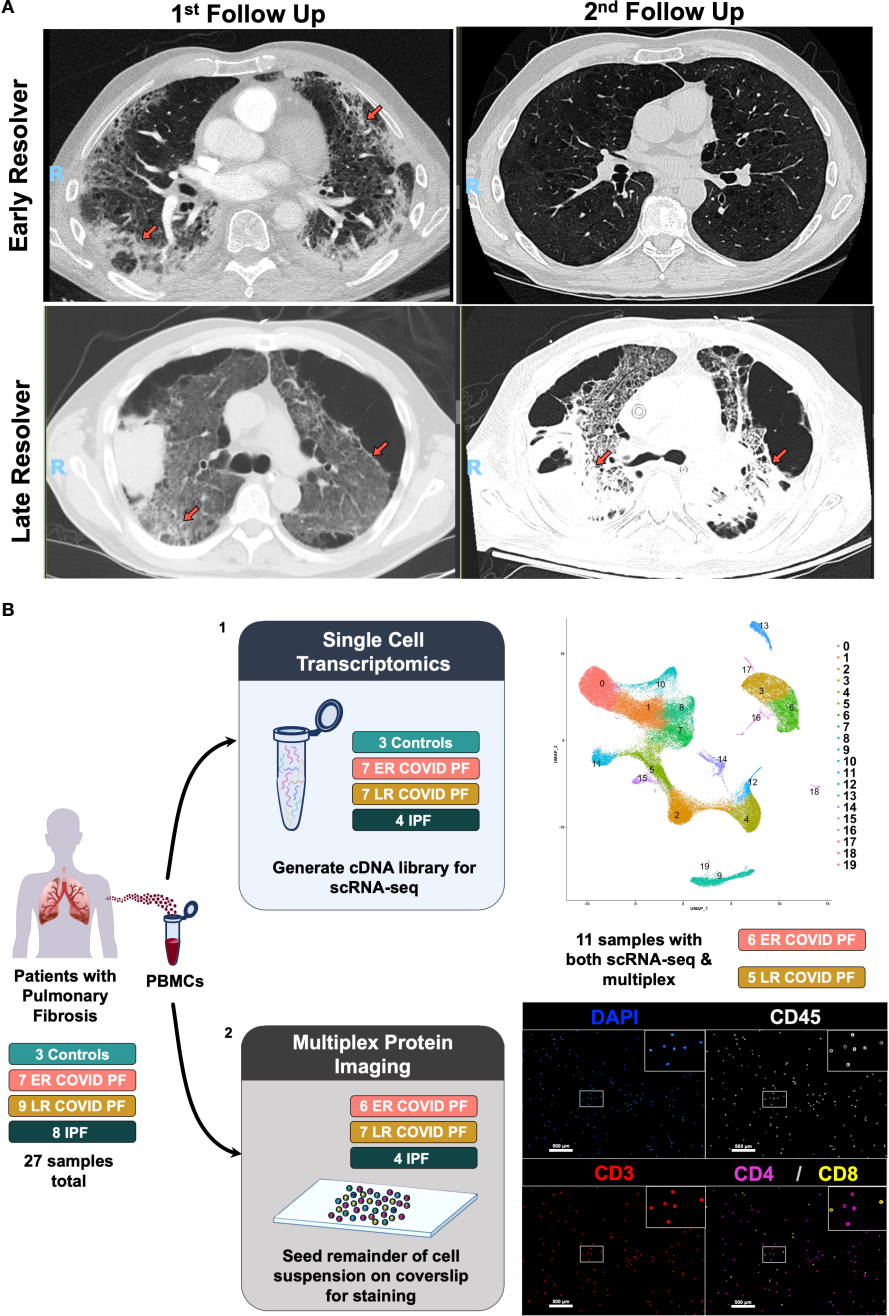
Computerized tomography images of Early- and Late- Resolving COVID associated pulmonary fibrosis and study design. **(A)** CT images of two COVID survivors displaying resolving pulmonary fibrosis (top row) or persistent pulmonary fibrosis (bottom row). Left images represent abnormal CT findings observed when first evaluated in the post-COVID clinic more than one month post-infection. Right images denote either the resolution or persistence of abnormal findings 6 or more months after infection. Abnormal findings such as ground glass opacities and reticulation are indicated by orange arrows. **(B)** Schematic of multi-omic study design where (1) depicts scRNA-seq processing and preliminary UMAP, and (2) depicts multiplex imaging workflow and representative image of stained PBMCs using PhenoCycler to identify T cells.

Here, we identify COVID-19 patients with restrictive lung physiology and early CT scan changes consistent with fibrosis more than one month after acute SARS-CoV-2 infection symptoms had resolved. At the second outpatient follow-up, the cohort diverges into two groups: Patients whose restriction and early imaging changes resolved, which we termed “Early-Resolvers” (ER COVID-PF), and those with persistent restriction and pulmonary fibrosis, whom we termed “Late-Resolvers” (LR COVID-PF) (**Figure 1A**). The objective of our study was to first define immune features that discriminated LR COVID-PF from ER COVID-PF by performing single-cell RNA sequencing and multiplex immunostaining analysis of PBMCs, and to second compare the resultant cellular and molecular signatures with IPF.

## Materials and methods

### Study participants

Patients with IPF donated peripheral blood mononuclear cells (PBMCs) in the outpatient setting while in a stable clinical state (IRB #20937). Three control PBMC samples from age-matched patients with no known pulmonary disease were prepared and sequenced at the Mayo Clinic (IRB #:19-012187). We recruited a subset of patients from the University of Virginia COVID-19 survivor clinic (IRB #:13166) with restrictive lung physiology on pulmonary function tests (PFTs) and features consistent with pulmonary fibrosis on chest CT performed at the associated visit (27). Radiographic features indicative of possible development of pulmonary fibrosis included bilateral reticulation, traction bronchiectasis, and/or honeycomb change in peripheral and basilar distribution, similar to the presently recognized progressive pulmonary fibrosis clinical radiologic phenotype, and similar to previously defined COVID-19 pulmonary fibrosis characteristics (7). PFT and chest imaging was performed in association with the patient’s first visit to the outpatient COVID-19 survivor clinic. Early- and Late-Resolvers were identified by comparing chest imaging and PFT values from the patient’s first and subsequent visit. Patients with COVID-19 associated pulmonary fibrosis (COVID-PF) were followed for 6 months or until the patient clinically improved. COVID-PF patients were age-matched to IPF patients to control for age-related differences in peripheral immune signatures.

### Sample collection

Patients who had IPF or had recovered from COVID-19 were recruited through the University of Virginia (UVA) Pulmonary and Post-COVID clinic respectively. PBMCs were isolated from venous blood (Post-COVID and IPF: K2EDTA BD Vacutainer®) by density gradient centrifugation and cryopreserved for later analysis (FBS + 10% DMSO). PBMC collection and single cell RNA sequencing for control samples were prepared at the Mayo Clinic similarly to samples detailed by Cheon et al. (16).

### Single cell RNA sequencing sample preparation

Single-cell RNA sequencing (scRNAseq) was performed on cryopreserved PBMCs from four IPF and fourteen COVID-19 associated pulmonary fibrosis (COVID-PF) patients at the University of Virginia. All analyses were conducted on COVID-PF PBMC samples collected at the COVID patient’s initial out-patient visit. Peripheral blood samples were prepared using the 10x Genomics Fresh Frozen Human PBMC protocol. Viability of thawed cells prior to sequencing was assessed using either a hemocytometer or Countess 3 (**Supplementary Table E3**). The generation of single cell indexed libraries, from the thawed PBMCs, was performed by the School of Medicine Genome Analysis and Technology Core, RRID : SCR_018883, using the 10X Genomics chromium controller platform and the Chromium Single Cell 5′ Library & Gel Bead Kit v1.1 reagent. Briefly, around 10,000 cells were targeted per sample and loaded onto each well of a Chromium Single Cell G Chip to generate single cell emulsions primed for reverse transcription. After breaking the emulsion, the single cell specific barcoded DNAs were subjected to cDNA amplification and quality control on the Agilent 4200 TapeStation Instrument, using the Agilent D5000 kit. Each sample cDNA was used to prepare indexed libraries that were pooled prior to sequencing. A quality control run was performed on the Illumina Miseq using the nano 300 cycle kit (1.4 Million reads/run), to estimate the number of targeted cells per sample. The cell estimate enabled the core to re-balance the pooled sample prior to deep sequencing onto either the NextSeq 500 using the 150 cycle kit or NextSeq 2000 using the P3-100 cycle kit. After run completion, the Binary base call (bcl) files were converted to fastq format using the Illumina bcl2fastq2 software, and data transferred to the Bioinformatics core. Data was aligned and quantified using the Cellranger 6.0.1 function except for the initial sample processed with Cellranger 4.0.0.

To accommodate batch effects, all submissions were prepared with samples from each of the different treatments (ER COVID-PF, LR COVID-PF, and IPF). This technique was successful for the vast majority of our data with the exception of the 3 non-diseased controls prepared at the Mayo Clinic (sequenced on MGISEQ-2000) and 3 of the 18 single-cell RNA seq samples prepared at UVA where the complementary samples failed quality control for that batch.

### Single cell RNA sequencing data cleaning and integration

Raw fastq files from all 21 samples were aligned to the GRCh38 human reference genome and quantified. Following the suggested pipeline for quality control in the Seurat v.4.0.3 package, sc-RNAseq data were filtered for dead cells, doublets, and red blood cells by excluding cells with greater than 5% mitochondrial genes and less than 500, but no more than 2500 genes. Samples underwent normalization, scaling, integration using anchors, dimensional reduction, and further downstream analysis using the standard Seurat workflow with the Seurat v.4.0.3 package. Principle components were visualized using an elbow plot to select dimensionality of the 21-sample integrated dataset. From this quantitative approach, we determined to implement 17 dimensions as our input for the RunUMAP and FindNeighbors clustering parameters and set the resolution to 0.5 for our initial clustering. Nineteen cell clusters were identified initially. Erythrocyte clusters were removed, and two CD4+ T effector cell clusters were merged, yielding 16 distinct immune cell subpopulations (69,868 cells). Dot plots depicting the defining markers for each cluster were constructed using default settings in the DotPlot function.

### Single cell RNA sequencing cell abundance quantification

Labels denoting each condition were appended to each cluster ID and counted using the Idents function in Seurat. These values were then exported to Excel where relative cell abundance to the total cell population were calculated ((number of cells in cluster x/sum of cells from all clusters)*100). Total cell population was replaced with all CD4 T cells or all CD8 T cells when applicable for T cell subset relative abundances. All bar charts representing relative cell abundance were constructed and statistically analyzed in GraphPad Prism 9.4.1. To determine whether the time between initial COVID infection and sample collection (in months) was a potential cofounding variable for cell abundance Pearson or Spearman Correlations (depending on normality determined by Shapiro-Wilk Test) were performed.

### Differentially expressed gene and gene set enrichment analyses

Differentially expressed genes (DEGs) between conditions (non-diseased control, ER COVID-PF, LR COVID-PF, and IPF) were determined using the FindMarkers function in Seurat. Significance for the difference in gene expression between the two groups were tested using the default Wilcox rank sum test and adjusted with bonferroni correction using all genes to account for false discovery. Violin plots depicting DEGs were constructed using default settings in the VlnPlot function. To account for time between initial COVID infection and sample collection (in months) as a potential cofounding variable we performed a multiple linear regression analysis using the latent.vars argument in the FindMarkers function.

Differentially expressed genes for gene set enrichment analysis (GSEA) were determined by applying model-based analysis of single-cell transcriptomics (MAST) test with Bonferroni adjustment and the clusterProfiler package to the whole gene expression profile of ER versus LR COVID-PF as well as each LR COVID-PF versus the non-diseased control group. The canonical pathways from the curated gene set (C2) provided by Molecular Signatures Database (MSigDB) were input as the gene list for GSEA. GSEA results were output as a table found in supplemental or visualized using the dotplot function from ggplot package.

Similarly, MAST DEG outputs were used to generate Volcano Plots using the EnhancedVolcano and tidyverse packages.

### Single cell RNA sequencing re-clustering

All CD4+ T cell populations were merged into one cluster and subset out for CD4+ T cell re-clustering. The subset object containing all CD4+ T cells was then re-processed for variable features using the vst selection method and setting nfeatures to 2000, followed by re-scaling in Seurat. For dimensional reduction, the dimension argument was set to 8 for the FindNeighbors function and the resolution to 0.75 for the FindClusters function. Nine populations were output from this analysis. Clusters 0 and 7 expressed similarly high levels of the naïve markers CCR7+ and SELL+ without expressing effector markers and therefore were combined into one Naïve CD4+ T cell population. Similarly, clusters 5 and 1 were combined as these clusters expressed lower levels of CCR7 and SELL while expressing similar amounts of the memory markers CD27, S100A4, and PASK. We identified a naïve subpopulation, an Early Activation subpopulation (denoted by high expression of naïve markers with moderate expression of activation markers, such as CD69+, suggesting these naïve cells were recently stimulated and in the early response of transitioning to an effector or memory function), Th1-like (TBX21+, CXCR3+), Th2-like (GATA3hi, CCR4+), Th17-like (RORC+, CCR6+), and Treg (FOXP3+, IL2RA+).

### Sample multiplex immunostaining

Protein expression was measured using the multiplex imaging platform PhenoCycler (Akoya Biosciences) according to the manufacturer’s protocol as it can utilize samples with low cell count. Six ER COVID-PF, seven LR COVID-PF, and four IPF samples were immunostained and quantified. Of those, six ER COVID-PF and five LR COVID-PF were also processed for sc-RNA-seq. Briefly, PBMCs from the same suspension processed for scRNA-seq were spun down at 300 rcf for 5 minutes and washed in Hydration Buffer (Akoya). Cells were then resuspended in 1.6% PFA diluted in Hydration Buffer and fixed for 20 minutes at room temperature on a rotator. The sample was then spun down and washed in Hydration Buffer again as stated above. Once the supernatant was removed, fixed PBMCs were then seeded onto a poly-L-lysine coated coverslip and allowed to air dry for 10 minutes. Dried coverslips were washed in PBS two times, incubated with Staining Buffer (Akoya) for 20 minutes, and then placed in a humidity chamber for staining with the 13-antibody panel. All samples were stained for three hours at room temperature in the humidity chamber with CD45 (Catalog # 4150003), CD2 (Catalog # 4250005), CD19 (Catalog # 4350003), CD38 (Catalog # 4150007), CD11c (Catalog # 4350012), CD278 (Catalog # 4250013), CD8 (Catalog # 4150004), CD3 (Catalog # 4350008), CD69 (Catalog # 4250022), CD4 (Catalog # 4350010), Ki67 (Catalog # 4250019), CD279 (Catalog # 4250010), and HLA-DR (Catalog # 4250006) using Akoya manufacturer’s instructions and blockers (Further information on antibodies in **Supplementary Table E2**). Coverslips were then washed in Staining Buffer two times, followed by a post-staining fixation with 1.6% PFA in Storage Buffer (Catalogue # 232107) for 10 minutes at room temperature. Slides were washed three times in PBS and incubated in ice cold methanol for 5 minutes. Samples were washed three times with PBS and then prepared for a final fixation in fresh BS3 diluted in PBS for 20 minutes before being washed in PBS three times and stored at 4 degrees C in Storage Buffer until imaging.

### Multiplex imaging and processing

Samples stained with the 13-antibody immune panel underwent multiplexed imaging using the spatial-omics platform PhenoCycler (Akoya) in combination with the BZ-X810 slide scanning microscope (Keyence). Further detail on automated imaging acquisition and fluidics exchange using the PhenoCycler is described by Goltsev et al. and Schürch et al. (28, 29). Raw TIFF images were stitched and processed using the PhenoCycler Processor which executes drift compensation, deconvolution, background subtraction, cycle alignment, and cell segmentation via a watershed cell segmentation algorithm. Data was then visualized and analyzed in MAV (Multiplex Analysis Viewer), a FIJI/ImageJ plugin.

Flow cytometry standard (FCS) files were generated from the processed data for each sample and imported to FCS Express 7 for further analysis.

### Quantification of protein expression and cell gating

**Figure 2:**
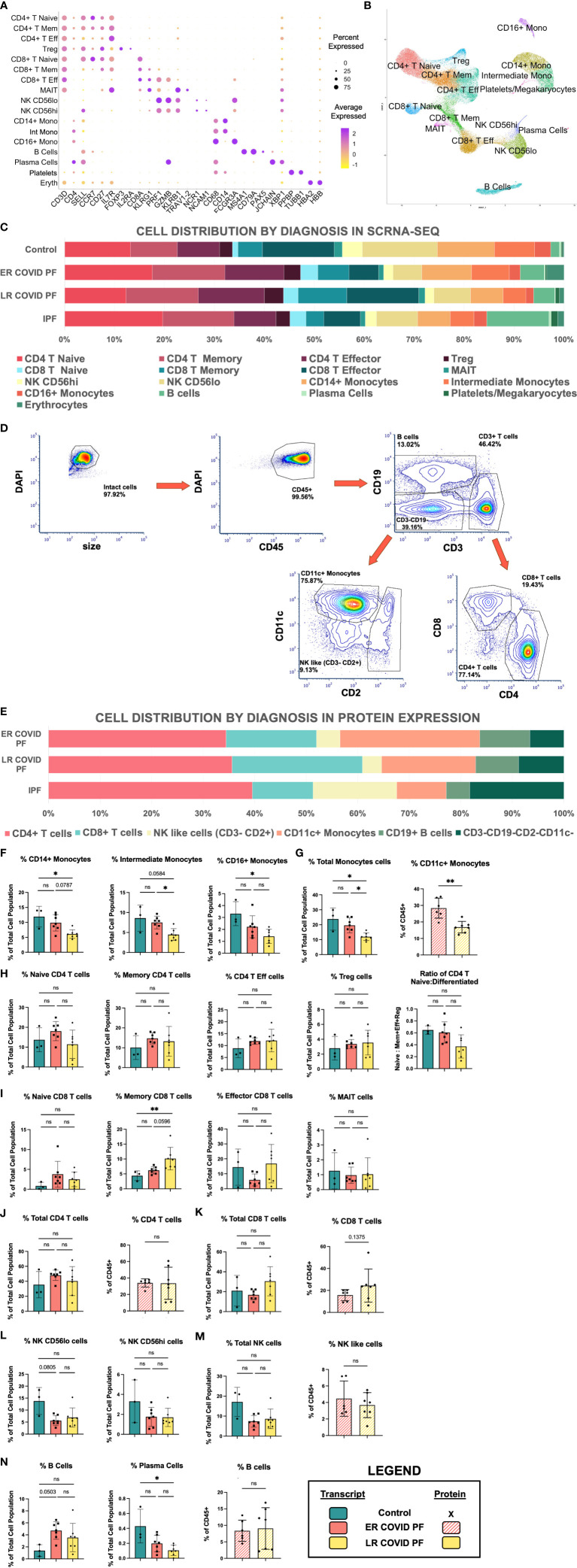
Cell abundances vary between ER and LR COVID-PF. **(A)** Dot plot denoting expression of marker genes used to identify 16 PBMC subpopulations as well as platelets and erythrocytes. **(B)** UMAP of cell clusters from integrated data of control, COVID, and IPF PBMCs generated through scRNA-seq in Seurat (21 samples total). **(C)** Stacked bar chart depicting relative cell abundances within each group (Control, ER COVID-PF, LR COVID-PF, and IPF) for the 16 subclusters identified by scRNA-seq. **(D)** Representative gating strategy used to identify 6 subpopulations from PBMCs immunostained on PhenoCycler. **(E)** Stacked bar chart depicting relative cell abundances within each treatment (ER COVID-PF, LR COVID-PF, and IPF) for the 6 subpopulations identified by immunostaining and imaging with the PhenoCycler. **(F)** ScRNA-seq relative abundances of monocyte subpopulations in Control, ER COVID-PF, and LR COVID-PF. **(G)** Relative abundance of total monocyte cell identified by scRNA-seq, left (solid), and protein on PhenoCycler, right (striped). **(H)** ScRNA-seq relative abundances of CD4+ T cell populations and ratio of naïve to differentiated (defined as the sum of effector, memory, and regulatory cells) in Control, ER COVID-PF, and LR COVID-PF. **(I)** ScRNA-seq relative abundances of CD8+ T cell populations in Control, ER COVID-PF, and LR COVID-PF. Relative total abundances of CD4+ T cells **(J)** and CD8+ T cells **(K)** identified by scRNA-seq, left (solid bar chart), and protein on PhenoCycler, right (striped bar chart). **(L)** ScRNA-seq relative abundances of natural killer subpopulations. **(M)** Relative abundance of total natural killer cell population identified by scRNA-seq, left (solid), and protein on PhenoCycler, right (striped). **(N)** ScRNA-seq relative abundances of B and plasma cell subpopulations (solid) and protien quantification of total CD19+ B cell population on PhenoCycler, right (striped). Transcript data acquired through scRNA-seq is displayed as solid bar charts, while protein data acquired through multiplex imaging on the PhenoCycler are displayed as striped bar charts. Kruskall-Wallis test with Dunn’s multiple comparison test was used to test significance when comparing ER COVID-PF, LR COVID-PF, and non-diseased control in *F-N*. Mann-Whitney U test was used when comparing two groups (ER COVID-PF and LR COVID-PF). *p ≤ 0.05, and ** p ≤ 0.01. All non-significant values p ≤ 0.1 are shown.*PBMC, peripheral blood mononuclear cell; scRNA-seq, single cell RNA sequencing; ER COVID-PF, “Early-Resolvers”; LR COVID-PF, “Late Resolvers”; IPF, Idiopathic Pulmonary Fibrosis; NK, Natural Killer Cells; FVC, Forced Vital Capacity; DLCO, Diffusing Capacity for Carbon Monoxide; TLC, Total Lung Capacity; Eff, Effector; Mem, memory; Reg, regulatory.

FCS files for each image region generated by the PhenoCycler were concatenated by staining batch and analyzed in FCS Express 7. Gates to determine cell type and protein expression were tailored to each sample in a blinded fashion by experienced flow cytometrist. Samples were gated on DAPI and CD45 double positive cells to identify nucleated PBMCs. Gating strategy for determining major cell types (CD4+ T cell, CD8+ T cell, CD11c+ Monocytes, CD3-CD2-CD19-CD11c- Natural Killer-like cells) is shown in **Figure 2D**. Gates to quantify percent of cells expressing HLA-DR, CD69, CD38, ICOS, PD-1, and Ki-67 were kept consistent when possible for each imaging batch, and known negative cell populations were used as an internal control, where possible. Signal and gating were confirmed by referencing processed images in MAV. All bar charts representing relative cell abundance and protein expression were constructed in GraphPad Prism 9.4.1.

### Statistics

Details regarding statistical tests used for each analysis are included above or in the respective figure caption. All statistical tests were performed in R Studio 4.1.1 or GraphPad Prism 9.4.1. Fisher’s Exact test was performed to evaluate whether there was a significant difference between sex and ethnicity of all COVID associated pulmonary fibrosis patients and Idiopathic Pulmonary Fibrosis. Mann-Whitney U test was used to compare Early- and Late-Resolving COVID associated pulmonary fibrosis hospital length of stay. For relative cell abundances and protein expression of specific markers significance was tested using Mann Whitney U test when comparing two groups, and Kruskal-Wallis test with Dunn’s multiple comparison when comparing three groups. Pearson correlation was used to determine significant correlations between PFT values and relative cell abundance. For genes displayed as violin plots, significance of the differentially expressed genes between two groups were tested using the default Wilcoxon rank sum test and adjusted with Bonferroni correction. For genes displayed on volcano plots the adjusted p value was calculated through MAST analysis with Bonferroni correction.

### Study approval

All studies performed at UVA were approved by the UVA Human Investigations Committee (IRB-HSR 13166, IRB-HSR 20937). Studies performed at the Mayo Clinic were approved by Mayo Clinic Institutional Review Boards (protocol ID 20-004911). All subjects provided written informed consent.

## Results

### COVID associated pulmonary fibrosis, idiopathic pulmonary fibrosis, and control cohort clinical features

To examine the immune response of patients with COVID associated PF, we recruited 16 patients without pre-existing PF. All had dyspnea, fatigue and abnormal lung function at their first outpatient visit after acute COVID-19 recovery. 14 were hospitalized and received: mechanical ventilation (n=10, mean time 30 ± 18 days), non-invasive positive pressure ventilation (n=1), high-flow nasal cannula oxygen (n=3) and 3 remained on outpatient supplemental oxygen. Hospital length of stay was prolonged (**Table 1**; **Supplementary Table E1**). Mean time since testing positive for COVID-19 was 6 ± 3.5 months.

**Table 1:** COVID associated pulmonary fibrosis and idiopathic pulmonary fibrosis cohort characteristics.

	Early Resolving COVID PF	Late Resolving COVID PF	IPF	p-value
	*n*=7	*n*=9	*n*=8	
Sex				>0.999
Male	6	7	7	-
Female	1	2	1	-
Ethnicity				0.624
White	6	6	7	-
African American	1	2	1	-
Native American	0	1	0	-
Age (years)	56 [52, 62]	59 [54, 68]	64 [63, 72]	0.1027
Body Mass Index (kg/m2)	36 [30, 40]	35 [30, 35]	34 [29, 37]	0.9723
FVC (% predicted)	79 [67, 94]	63 [59, 73]	62 [55, 80]	0.1466
DLCO (% predicted)	67 [57, 95]	56 [52, 67]	42 [38, 60]	0.1079
TLC (% predicted)	76 [63, 87]	64 [59, 74]	59 [54, 73]	0.1074
Hospital Length of Stay (days)	19 [4, 19]	38 [30, 54]	*NA*	0.0209

PF, pulmonary fibrosis; IPF, Idiopathic Pulmonary Fibrosis; FVC, forced vital capacity; DLCO, diffusing capacity of lung for carbon monoxide; TLC, total lung capacity; NA, not applicable.

Table detailing clinical features of early and late resolvers within COVID associated pulmonary fibrosis patients with Idiopathic Pulmonary Fibrosis (IPF). Data shown are displayed as mean [Q1, Q3] or raw numbers for sex and ethnicity. Two patients were unable to perform TLC testing on draw date and were therefore not incorporated into statistics. Fisher’s Exact test was performed to evaluate whether there was a significant difference between sex and ethnicity of all COVID associated pulmonary fibrosis patients and Idiopathic Pulmonary Fibrosis. Mann-Whitney U test was used to compare Early- and Late-Resolving COVID associated pulmonary fibrosis hospital length of stay. Kruskal-Wallis test was performed on all other values to test whether there was a significant difference between Idiopathic Pulmonary Fibrosis, Early- and Late- Resolving COVID associated pulmonary fibrosis.

At mean follow-up 5.4 months, 9 patients showed a late recovery trajectory which presented a unique opportunity to investigate the resolution and persistence of COVID associated PF (**Figure 1A**). There was no significant difference in age, sex, body mass index (**Table 1**) or medical comorbidities between ER and LR COVID-PF. 89% of LR and 29% of ER COVID-PF patients underwent mechanical ventilation. 86% of ER COVID-PF had normalized PFTs at mean 9.5 months after testing positive for COVID-19. 33% of LR COVID-PF patients normalized, while the majority had persistent restrictive lung physiology at mean 11 months after testing positive for COVID-19 and 7 months after hospital discharge. All LR COVID-PF had chest imaging showing persistent bilateral fibrotic change.

8 IPF patients comparable in age were also selected as a positive control of chronic progressive pulmonary fibrosis. Mean age was 64, mean BMI was 34, and 7 of the 8 patients were men, matching the demographics and body mass of the COVID-19 cohort (**Table 1**; **Supplementary Table E1**). PFTs measured at their clinically stable outpatient visit were mean forced vital capacity (FVC) 62% predicted and diffusion capacity (DLCO) 42% predicted. Six used supplemental oxygen. In addition, 3 age-matched patients without known pulmonary disease were sequenced as non-pulmonary disease controls (Mean age = 69, All male).

### Single-cell RNA sequencing and multiplex immunostaining

To profile the peripheral immune response in post-COVID pulmonary fibrosis, we generated two rich and complementary datasets using PBMC samples collected at the COVID-19 patients’ first outpatient visit after COVID recovery (**Figure 1B**). For in-depth characterization of transcriptional differences, we performed scRNA-seq on 21 subjects (seven ER COVID-PF, seven LR COVID-PF, four IPF, and three controls) and integrated the data to yield a combined 71,574 cells (**Supplementary Figure 1B1**). Dimensionality reduction by uniform manifold approximation and projection (UMAP) identified 19 cell clusters. We removed erythrocytes and merged two CD4+ T effector cell clusters to yield 16 distinct immune subpopulations (69,868 cells), which were identified and annotated using established markers (**Figures 2A, B**).

To complement and validate this sequencing dataset with protein-level data, we generated a 13-antibody panel (**Supplementary Figures 1B2, E1**; **Supplementary Table E2**) and immunostained PBMC samples from 17 subjects (six ER COVID-PF, seven LR COVID-PF, and four IPF). After blinded gating, B cell, Monocyte, NK-like, CD4+ and CD8+ T cell subpopulations were identified (**Figure 2D**). Eleven COVID samples underwent both scRNA-seq and multiplex immunostaining (**Supplementary Table E3**).

All major subpopulations identified by immunostaining and all 16 subpopulations identified by scRNA-seq were present in each sample, indicating data consistency (**Figures 2C, E**; **Supplementary Figure E2**). These datasets enabled comparisons of immune cell abundance and gene expression of each cell type among ER and LR COVID-PF, IPF and controls.

### Cell abundance varies between early- and late-resolving COVID-PF

First, we examined whether the relative abundances of circulating immune cells differ in ER and LR COVID-PF (**Figures 2F–N**). Compared to controls, LR COVID-PF had significantly fewer plasma cells (p = 0.0231, **Figure 2N**). There were no differences in NK like cells or CD4+ T cell abundance between groups, however the ratio of naïve to differentiated (where CD4 differentiated is the sum of regulatory, memory, and effector subpopulations) decreased in the LR COVID-PF (p=0.1252). LR COVID-PF had increased CD8+ T cells compared to ER COVID-PF, showing a 1.6-fold increase in CD8+ T memory cells. Within LR COVID-PF, there were no significant but trending increases in effector and total CD8+ T cells at the transcript and protein level (**Figures 2I, K**).

Notably, LR COVID-PF showed significant decreases in all monocyte populations compared to ER COVID-PF and controls (**Figures 2F, G**). For rigor, we tested whether time from initial COVID positivity to sample collection was correlated with monocyte abundance and found no correlation (**Supplementary Table E4**). Quantification of cellular abundance by multiplex immunostaining corroborated that relative abundance of CD11c+ monocytes to all PBMCs is significantly lower in LR versus ER COVID-PF (p = 0.0023, **Figure 2G**).

### Circulating monocyte depletion associates with decreased pulmonary function in COVID-PF

**Figure 3:**
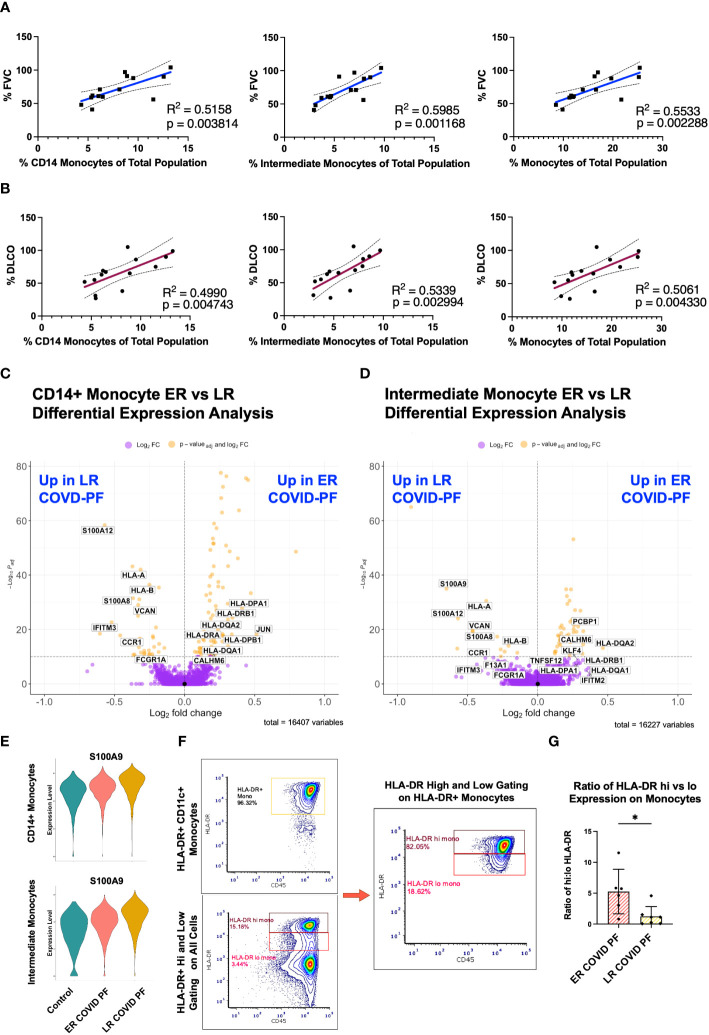
Decreased monocyte abundance correlates with decreased lung function in COVID associated pulmonary fibrosis. Pearson correlation of % Forced Vital Capacity (FVC) **(A)** and % Diffusing Capacity for Carbon Monoxide (DLCO) **(B)** compared to relative abundance of CD14+, intermediate, and total monocytes of ER and LR COVID-PF patients from scRNA-seq dataset. Volcano plot showing differentially expressed genes between ER and LR COVID-PF generated from MAST analysis for CD14+ monocytes **(C)** and intermediate monocytes **(D)** where positive log2FC values represent genes upregulated in ER COVID-PF relative to LR COVID-PF and negative log2FC represent genes upregulated in LR COVID-PF relative to ER COVID-PF. Y axis of the volcano plot is the -log_10_ of the p_adj_-value calculated using Bonferroni correction to correct for multiple testing **(E)** Violin plots showing gene expression of the alarmin, S100A9, between control, ER COVID-PF, and LR COVID-PF in CD14+ and Intermediate Monocytes. Expression of S100A9 in CD14+ and Intermediate Monocytes of LR COVID-PF is significantly lower than control and ER COVID-PF where p_adj_-value ≤ 1E-40 as determined by non-parametric Wilcoxon rank sum test and adjusted using Bonferroni correction. **(F)** Representative gating strategy used for quantifying high and low expression of HLA-DR on monocytes. **(G)** Quantification of the ratio of high to low expression of HLA-DR among HLA-DR+ monocytes at the protein level from imaging with the PhenoCycler. Significance for protein quantification in **(G)** was tested using Mann-Whitney U test. *p ≤ 0.05.

The decrease in relative monocyte abundance within LR COVID-PF was the only abundance difference found to be significant at both the transcript and protein level. We therefore next determined whether monocyte abundance correlates with pulmonary function in COVID-PF. The relative abundances of CD14+ monocytes, intermediate monocytes (CD14+ CD16+), and total monocytes each positively correlated with both FVC (% predicted FVC, R^2 = ^0.52, 0.60, 0.55, respectively) and DLCO (% predicted DLCO, R^2 = ^0.50, 0.53, 0.51, respectively) (**Figures 3A, B**).

To evaluate whether there were differences in the transcriptome of ER and LR COVID-PF monocytes, differential expression analysis was performed. Alarmins, such as S100A12, S100A9, and S100A8, were among the most significantly enriched genes within LR COVID-PF monocytes (**Figure 3E**). Differential expression analysis also revealed downregulation of MHC class II molecules, such as HLA-DPA1, HLA-DPB1, HLA-DRB1, and HLA-DRA, in circulating CD14+ and intermediate monocytes from LR COVID-PF in comparison to ER COVID-PF and controls (**Figures 3C, D**; **Supplementary Tables E5, 6**). Consistent with these transcriptomic results, the ratio of high to low HLA-DR protein expression on monocytes revealed a four-fold decrease (p = 0.0221) in LR versus ER COVID-PF (**Figures 3F, G**). Our data are consistent with previous studies showing increased alarmins and decreased MHC-II molecule expression on monocytes in severe versus mild COVID-19 in acute cases (12, 30–34), while also establishing that these markers can differentiate post-acute COVID-19 recovery trajectory including COVID-PF.

### Late-resolving COVID associated pulmonary fibrosis patients show prolonged CD8+ T cell activation and increases in CD4+ T effector populations

**Figure 4:**
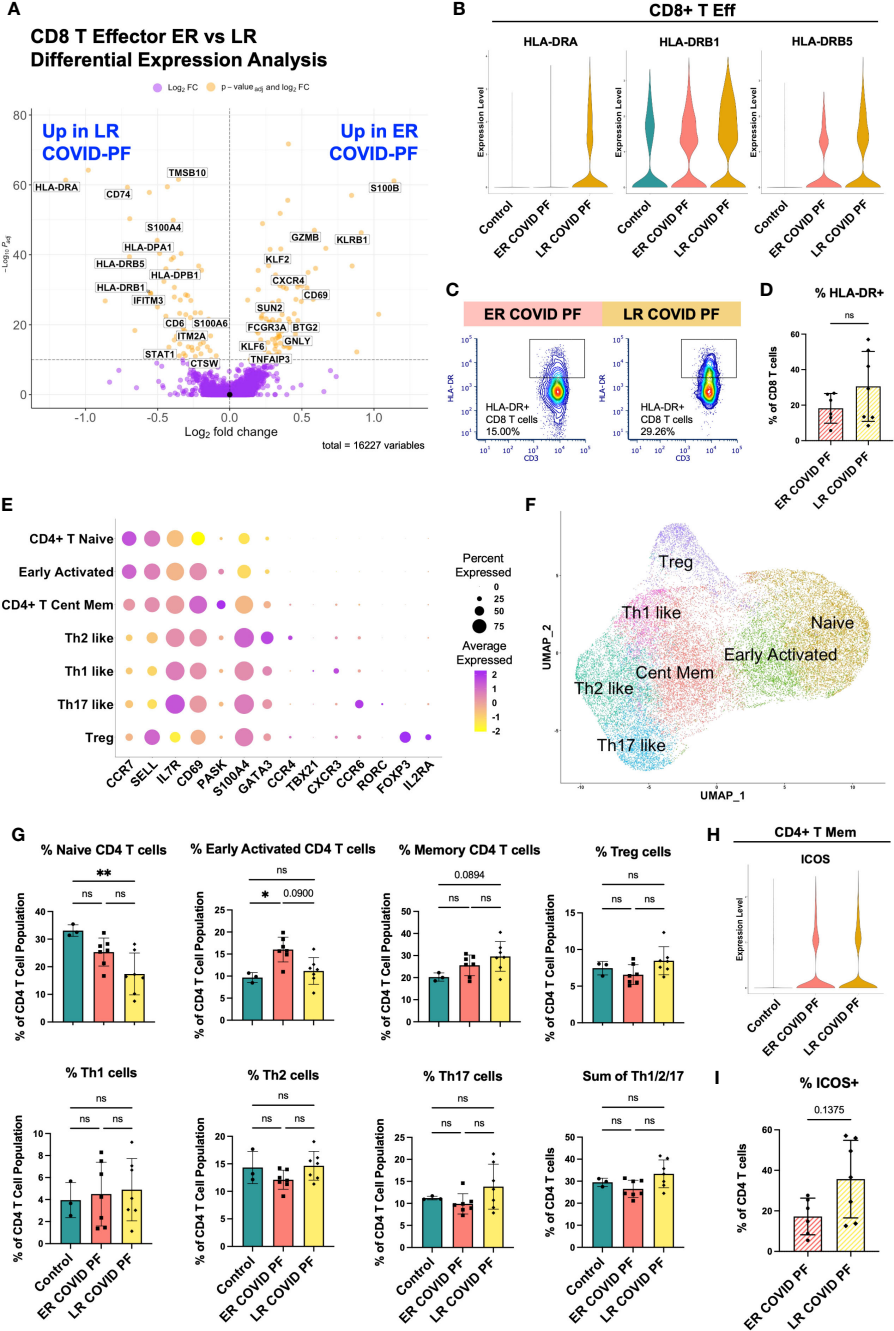
Late-Resolving COVID associated pulmonary fibrosis exhibits molecular hallmarks of prolonged T cell activation. **(A)** Volcano plot showing differentially expressed genes between ER and LR COVID-PF generated from MAST analysis for CD8+ T effector cells where positive log2FC values represent genes upregulated in ER COVID-PF relative to LR COVID-PF and negative log2FC represent genes upregulated in LR COVID-PF relative to ER COVID-PF. Y axis of the volcano plot is the -log_10_ of the p_adj_-value calculated using Bonferroni correction to correct for multiple testing. **(B)** Violin plots showing gene expression of MHC-II molecules in CD8+ T effector cells between control, ER COVID-PF, and LR COVID-PF. Expression of HLA-DRA, HLA-DRB1, and HLA-DRB5 in LR COVID-PF CD8+ T effector cells is signifcantly lower than control and ER COVID-PF where p_adj_-value ≤ 1E-26 as determined by non-parametric Wilcoxon rank sum test and adjusted using Bonferroni correction. **(C)** Representative gating of HLA-DR expression on CD8+ T cells. **(D)** Quantification of HLA-DR expression on CD8 T cells as a percent of CD8+ T cells at the protein level from imaging with the PhenoCycler in ER and LR COVID-PF. **(E)** Dot plot denoting expression of marker genes used to identify CD4+ T cell subpopulations. **(F)** UMAP of subclusters generated from re-clustering CD4+ T cells. **(G)** ScRNA-seq relative abundances of CD4+ T relative to the total CD4+ T cell population for control, ER COVID-PF, and LR COVID-PF. **(H)** Violin plot showing gene expression of ICOS in CD4+ T memory cells between control, ER COVID-PF, and LR COVID-PF. **(I)** Quantification of the percent of CD4+ T cells in ER and LR COVID-PF expressing ICOS at the protein level from imaging with the PhenoCycler. Transcript data acquired through scRNA-seq is displayed as solid bar charts, while protein data acquired through multiplex imaging on the PhenoCycler are displayed as striped bar charts. Mann-Whitney U test was used when comparing two groups in **(D, I)**. Kruskal Wallis test with Dunn’s multiple comparison test was used to test significance when comparing ER COVID-PF, LR COVID-PF, and non-diseased control in **(F)**.*p ≤ 0.05 and ** p ≤ 0.01. All non-significant values p ≤ 0.1 are shown.

To investigate whether T cell-associated mechanisms differentiated ER and LR COVID-PF, we identified unique transcript and protein signatures among lymphocytes. CD8+ T effector cells had the highest number of differentially expressed genes (DEGs) of all PBMC subpopulations, suggesting elevated activation status (**Supplementary Table E7**). Contrary to LR COVID-PF monocytes, HLA-DRA, HLA-DPA1, and HLA-DRB5 were among the top DEGs upregulated in LR COVID-PF CD8+ T effector cells compared to ER COVID-PF and controls (p_adj_-value<1.0E-10, Wilcoxon Rank Sum test with Bonferroni correction) (**Figures 4A, B**). These increases persisted when controlling for time from initial COVID positivity to sample collection as a confounding variable (**Supplementary Table E7**). Immunofluorescent staining confirmed CD8+ T cells of LR COVID-PF had a nearly 3-fold increase in HLA-DR protein expression (**Figures 4C, D**) and 4-fold increase in the proportion of CD8+ T cells that co-express HLA-DR+ and CD38+, a phenotype associated with lymphocyte activation (**Supplementary Figure E5**). Given that PBMCs were collected months after initial infection, these results suggest LR COVID patients exhibit prolonged immune activation, reminiscent of chronic inflammation.

To execute an unbiased analysis of the changes between ER and LR COVID-PF gene expression within CD4+ and CD8+ T effector cells, we implemented the Model-based Analysis of Single-cell Transcriptomics (MAST) test and clusterProfiler package to perform gene set enrichment analysis (GSEA) (35–37). In alignment with our hypothesis that LR COVID-PF exhibits prolonged T cell activation, eight T cell receptor signaling pathways were enriched in LR COVID-PF compared to controls (**Supplementary Tables E8**; **Supplementary Figure E4**). Furthermore, CD8+ T effector cells in both ER and LR COVID-PF were positively enriched for gene sets involving Proinflammatory and Profibrotic Mediators, Cytokine-cytokine Receptor Interactions, and the Network Map of SARS-CoV2 Signaling Pathway when compared to control. Within ER and LR COVID-PF, we also observed enrichment of multiple IL-1 and CD40 pathways as well as the Senescence Associated Secretory Phenotype (SASP). Notably, Allograft Rejection and Interferon Gamma (IFN-γ) Signaling were significantly enriched in LR COVID-PF compared to control and ER COVID-PF. This corroborates other studies which have found increased IFN-γ in severe COVID (30, 38, 39). Both CD8+ and CD4+ T effector cells in ER and LR COVID-PF were positively enriched for IL-10, IL-18, and multiple MAPK and Toll Like Receptor Signaling pathways compared to control (**Supplementary Table E8**). Interestingly, ER and LR COVID-PF were negatively enriched for Extracellular Matrix (ECM) Organization in CD8+ T effector cells and ECM Regulators in CD4+ T effector cells in comparison to control, suggesting that T cell mediated ECM regulation may be decreased in COVID-PF. Lastly, we observed upregulation of the TGF-β pathway, a key driver of fibrosis, in ER and LR COVID-PF CD4+ T effector cells (**Supplementary Table E8**).

To further identify classical T helper cell subtypes, we reclustered all CD4+ T cells, yielding seven distinct populations: Naïve, Early Activation (denoted by high expression of naïve markers with moderate expression of activation markers), Central Memory, and several distinct effector populations (Th1, Th2, Th17, and Treg) that expressed canonical markers (**Figures 4E, F**). Corroborating results in **Figure 2H**, LR COVID-PF patients had significantly fewer naïve CD4+ T cells than control (**Figure 4G**), and had a smaller Early Activated population compared to ER COVID-PF. In contrast, central memory, Treg, and effector (sum of Th1, Th2, and Th17) CD4+ T cells were increased in LR COVID-PF, although not significantly. These results suggest that the CD4+ T helper response in LR COVID-PF has been skewed to an active effector/memory phenotype. In support of this notion, we found an increase in the protein expression of the co-stimulatory molecule ICOS (**Figure 4I**), as well as increased protein expression of PD1 and CD69 on CD4+ T cells of LR COVID-PF patients compared to those of ER COVID-PF patients (**Supplementary Figure E3**). To determine which CD4+ T cell population was expressing ICOS, we queried our scRNA-seq data and detected ICOS on the CD4+ T memory population (**Figure 4H**). As ICOS is rapidly expressed after T cell receptor engagement and broadly expressed in activated T cells, this data aligns with our GSEA and CD4+ T cell abundance findings which suggested an activated phenotype within LR COVID-PF patients.

### Late-resolving COVID associated pulmonary fibrosis patients maintain a perpetual T cell activation response

**Figure 5:**
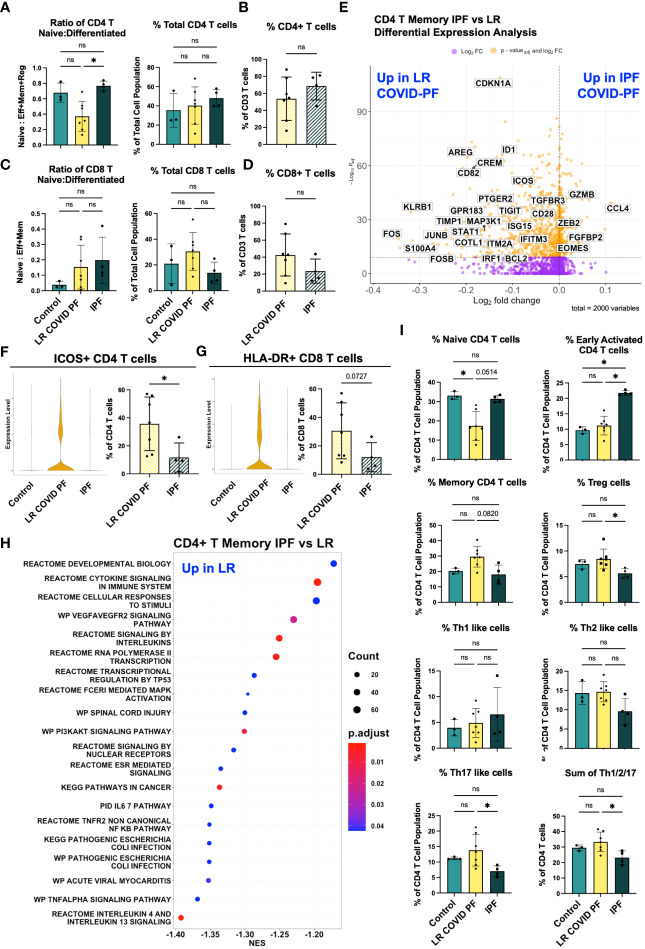
Adaptive immune cells differentiate IPF and COVID-PF. **(A)** ScRNA-seq data of CD4+ T cells showing the ratio of naïve to differentiated (defined as the sum of effector, memory, and regulatory cells) and relative total abundance of CD4+ T cells in Control, LR COVID-PF, and IPF. **(B)** Protein quantification of relative total abundance of CD4+ T cells in LR COVID-PF and IPF as a percent of all CD3+ T cells. **(C)** ScRNA-seq data of CD8+ T cells showing the ratio of naïve to differentiated (defined as the sum of effector and memory cells) and relative total abundance of CD8+ T cells in Control, LR COVID-PF, and IPF. **(D)** Protein quantification of relative total abundance of CD8+ T cells in LR COVID-PF and IPF as a percent of all CD3+ T cells. **(E)** Volcano plot showing differentially expressed genes between IPF and LR COVID-PF generated from MAST analysis for CD4+ T memory cells where positive log2FC values represent genes upregulated in IPF relative to LR COVID-PF and negative log2FC represent genes upregulated in LR COVID-PF relative to IPF. Y axis of the volcano plot is the -log_10_ of the p_adj_-value calculated using Bonferroni correction to correct for multiple testing. **(F)** Violin plots showing gene expression of ICOS in CD4+ T memory cells between control, ER COVID-PF, and LR COVID-PF and quantification of the percent of CD4+ T cells expressing ICOS at the protein level from imaging with the PhenoCycler. **(G)** Violin plots showing gene expression of MHC-II molecules (HLA-DR) in CD8+ T effector cells between control, ER COVID-PF, and LR COVID-PF and quantification of the percent of CD8+ T cells expressing HLA-DR at the protein level from imaging with the PhenoCycler. Gene expression of ICOS in LR COVID-PF CD4+ T memory cells and HLA-DRA in LR COVID-PF CD8+ T effector cells is signifcantly lower than control and ER COVID-PF where p_adj_-value ≤ 1E-14 as determined by non-parametric Wilcoxon rank sum test and adjusted using Bonferroni correction. **(H)** Dot plot depicting GSEA analysis results of the top 20 pathways enriched in LR COVID-PF compared to IPF, where negative NES are enriched in LR COVID-PF relative IPF. **(I)** ScRNA-seq relative abuduances of the CD4+ T cells relative to the total CD4+ T cell population for control, LR COVID-PF, and IPF. Transcript data acquired through scRNA-seq is displayed as solid bar charts, while protein data acquired through multiplex imaging on the PhenoCycler are displayed as striped bar charts. Kruskal Wallis test with Dunn’s multiple comparison test was used to test significance when comparing ER COVID-PF, LR COVID-PF, and non-diseased control in **(A, C, I)**. Mann-Whitney U test was used when comparing two groups in **(B, D, F, G)**. *p ≤ 0.05, and ** p ≤ 0.01. All non-significant values p ≤ 0.1 are shown.

We next evaluated whether immune signatures in LR COVID-PF had similarities to progressive pulmonary fibrosis. There was no significant difference in relative monocyte abundance (**Supplementary Figures E6B, C**), total number of T cells, or ratio of naïve to differentiated T cells in IPF compared to control (**Figures 5A–D**). The ratio of naïve to differentiated CD4 T cells was significantly lower in LR COVID-PF compared to IPF (p=0.0123). Re-clustering the CD4+ T cell dataset showed IPF patients had more naïve CD4 T cells and decreases in all effector cells, except Th1-like cells, compared to LR COVID-PF (**Figure 5I**).

To determine which genes were most significantly up or down-regulated in LR COVID-PF compared to IPF, we performed DEG analysis on all 16 scRNA-seq clusters. CD4+ T memory cells had the most DEGs. Genes involved in interferon signaling (e.g., IRF1, IFITM3, ISG15), highlighted as a key pathway in severe COVID (30, 38, 39), were upregulated in CD4+ T memory cells of LR COVID-PF (**Figure 5E**; **Supplementary Table E9**). In accordance with previous IPF literature (40–42), we find CD4+ T memory cells in IPF were characterized by gene expression patterns associated with senescence and exhaustion, such as ZEB2, EOMES, and decreased CD28 expression (43–45). Previously, loss of CD28 and ICOS correlated with reduced transplant-free survival in IPF (40). We observed reductions in both CD28 and ICOS in IPF compared to LR COVID-PF at the transcript level (**Figure 5E**). Expression of ICOS protein was also significantly greater (p=0.0424) in CD4+ T cells of LR COVID-PF compared to IPF, suggesting that T cell activation is enhanced in LR COVID-PF in comparison to control, ER COVID-PF, and IPF (**Figure 5F**). GSEA of CD4+ T memory cells in IPF versus LR COVID-PF further demonstrated that LR COVID-PF patients exhibit signs of potentiated inflammation as indicated by enrichment of TNFα, cytokine, and multiple interleukin signaling pathways (**Figure 5H**). Furthermore, LR COVID-PF had increased expression of HLA-DRA in CD8+ T effector cells (p_adj_-value=1.265E-14, Wilcoxon Rank Sum test with Bonferroni correction) and a significantly higher percentage of CD8+ T cells co-expressing HLA-DR+ and CD38+ (p=0.0273) when compared to IPF (**Figure 5G**; **Supplementary Figure E5F**). These data suggest T cells from LR COVID-PF patients are more active than their IPF counterparts, which express more senescence and exhaustion markers.

DEG analysis of monocytes revealed decreased expression of HLA-DR/DP on non-classical CD16+ monocytes in IPF (**Supplementary Tables E5, 6**), and on classical CD14+ and intermediate monocytes in LR COVID-PF (p_adj_-value<1E-7, **Supplementary Figure E6A**). These results suggest that reductions in monocyte HLA-DR are a common feature of PF.

## Discussion

We examined COVID-19 convalescent patients with persistent dyspnea and fatigue, abnormal PFTs and imaging suggestive of early pulmonary fibrosis. While some patients clinically improved in the outpatient setting in a matter of months (ER COVID-PF), others did not (LR COVID-PF). Here, we used single cell transcriptomics and a multiplex imaging approach to analyze blood samples collected more than one-month post-infection but before these two cohorts clinically diverged, and we uncovered that immune cell composition and gene expression significantly differed between ER and LR COVID-PF patients.

A key finding of this study is LR COVID-PF patients had significantly fewer monocytes than ER COVID-PF patients and controls. Our study is the first to identify that decreased relative monocyte abundance correlates with impaired pulmonary function in COVID-PF. These findings demonstrate that monocyte depletion not only distinguishes severe COVID from controls (31, 32, 46), but also has potential to stratify severity of COVID-19 sequelae. We hypothesize that in LR COVID-PF monocytes are either systemically depleted or alternatively recruited from the periphery to the lung or other tissues. Studies confirm increased infiltration of monocyte-derived macrophages in the bronchoalveolar lavage fluid of severe COVID-19 patients (47) and lungs of fatal COVID-19 (48). This observation suggests inhibiting monocyte recruitment may improve recovery from COVID-PF.

We also find monocytes of LR COVID-PF patients expressed lower levels of MHC class II molecules. CD16+ monocytes are more mature and express more HLA-DR than CD14+ monocytes, therefore reduction in HLA-DR+ CD14+ monocytes associates with mobilization of immature monocytes from the bone marrow for emergency myelopoiesis (49) which we observe here and others reported as a marker of severe COVID-19 (46, 50). Loss of HLA-DR on monocytes is also an established marker of immunosuppression, so these findings may also suggest dampening of antigen-mediated stimulation and inhibition of antigen-specific T cell responses as has been shown in sepsis (51–53). Aligned with this hypothesis, Arunachalam et al. reported functional suppression of COVID-19 monocytes compared to healthy controls (34). Decrease in the MHC class II molecule HLA-DR on monocytes also associates with severe respiratory failure in COVID-19 pneumonia (12), immunosuppression (54), and decreased oxygen saturation in severe COVID-19 (55). Of note, the cohorts in these studies included patients who were acutely infected, whereas our study shows that HLA-DR downregulation can be prolonged months after infection. Parackova et al. found that monocyte HLA-DR in COVID-19 patients began to recover four weeks into hospital admission (31). Thus, expression of HLA-DR on monocytes may indicate recovery, as the LR COVID-PF cohort had low HLA-DR expression more than 1-month post-infection, while patients with ER COVID-PF maintained or recovered HLA-DR expression.

Patients with IPF also display decreased expression of MHC-II molecules on monocytes relative to age-matched controls. Importantly, we demonstrate HLA-DR expression decreased exclusively on CD16+ monocytes in IPF, while conversely CD14+ and intermediate monocytes are the key populations affected in LR COVID-PF. It is possible that our results in IPF indicate immunoparesis, while in COVID-PF, enhanced migration of monocytes to the lung during COVID-PF may evoke emergency myelopoiesis and eventually promote a state of exhaustion. Whether the observed decrease in HLA-DR+ monocytes in IPF and LR COVID-PF arise from the same mechanism remains unknown, but further review of monocyte dysfunction in pulmonary fibrosis will require careful consideration of which monocyte subpopulations are affected.

Severe COVID-19 has been associated with increased CD8+ T cell activation (30, 56) and suppression of naïve CD4 T cells (8, 57–60). We observe both T cell phenotypes in LR COVID-PF. Compared to ER COVID-PF, controls, and IPF, LR COVID-PF had fewer naïve CD4+ T cells, and their CD8+ T cells expressed significantly greater levels of activation markers (HLA-DR and CD38). We therefore posit that the T cell response in LR COVID-PF is polarized toward an effector or memory phenotype rather than naïve state, extending the timeline of T cell abnormalities described in immediate post-acute COVID-19 (59). This chronic inflammatory state may lead to senescence of CD8+ T cells, a state common in IPF (40–42). In support of this notion, shortened telomere length, a defining characteristic of cellular senescence and feature associated with worse survival in IPF (61), was shown to be an independent risk factor for developing fibrotic-like radiographic abnormalities after severe COVID-19 (18). Future studies may explore whether monocytes in patients with LR COVID-PF have shortened telomeres and whether telomere length is a risk for LR COVID-PF.

Our cohorts were majority male to facilitate comparisons with our IPF cohort. However, this intentional gender composition decreases our capacity to draw comparisons between genders. Furthermore, the sample size was constrained by the unique nature of our cohort, and follow-up was confined to the first year after COVID-19 survivorship. Further studies in diverse large multicenter cohorts and analysis of immune signature changes in lung tissue are necessary for a more thorough understanding of the observed responses.

Post-COVID fibrosis is an emerging cause of global morbidity, and longitudinal studies are needed to evaluate the disease course in these patients. Our data mitigates fears that COVID-19 drives relentlessly progressive pulmonary fibrosis akin to IPF, even in the most persistently symptomatic COVID-19 survivors. Rather we demonstrate the peripheral immune response of LR COVID-PF is distinct from IPF, with decreases in HLA-DR expression on different monocyte populations. Our data provokes future studies of the systemic immune response in ER versus LR COVID-PF and identifies targets for future testing. We propose that relative monocyte abundance may be a clinically useful and simple prognostic indicator for determining whether long-haul COVID patients will resolve or have persistent pulmonary complications after acute COVID-19 recovery.

## Data availability statement

The datasets presented in this study can be found in online repositories. The names of the repository/repositories and accession number(s) can be found here: GSE249513 (GEO).

## Ethics statement

The studies involving humans were approved by the UVA Human Investigations Committee (IRB-HSR 13166 and IRB-HSR 20937) and the Mayo Clinic Institutional Review Boards (protocol ID 20-004911). The studies were conducted in accordance with the local legislation and institutional requirements. The participants provided their written informed consent to participate in this study.

## Author contributions

GB: Conceptualization, Data curation, Formal analysis, Investigation, Methodology, Visualization, Writing – original draft, Writing – review & editing. LM: Formal analysis, Project administration, Resources, Writing – review & editing. CL: Data curation, Writing – review & editing. YH: Formal analysis, Writing – review & editing. S-FM: Resources, Writing – review & editing. DA: Supervision, Writing – review & editing. IN: Supervision, Writing – review & editing. JS: Resources, Supervision, Writing – review & editing. JW: Resources, Supervision, Writing – review & editing. TB: Supervision, Writing – review & editing. CB: Conceptualization, Data curation, Investigation, Methodology, Resources, Supervision, Writing – review & editing.
