# Supplementary material for: High-dimensional comparison of monocytes and T cells in post-COVID and idiopathic pulmonary fibrosis
Source: Front Immunol. 2024 Jan 16;14:1308594. doi: 10.3389/fimmu.2023.1308594 (PMC10824838; doi:10.3389/fimmu.2023.1308594)
Supplement: Supplementary file 1 [file DataSheet_1.docx]

**High-dimensional comparison of monocytes and T cells in post-COVID and idiopathic pulmonary fibrosis**

**Authors:**

Grace C. Bingham, Lyndsey M. Muehling, Chaofan Li, Yong Huang, Shwu-Fan Ma, Daniel Abebayehu, Imre Noth, Jie Sun, Judith A. Woodfolk, Thomas H. Barker, and Catherine A. Bonham

**Supplemental Figures**


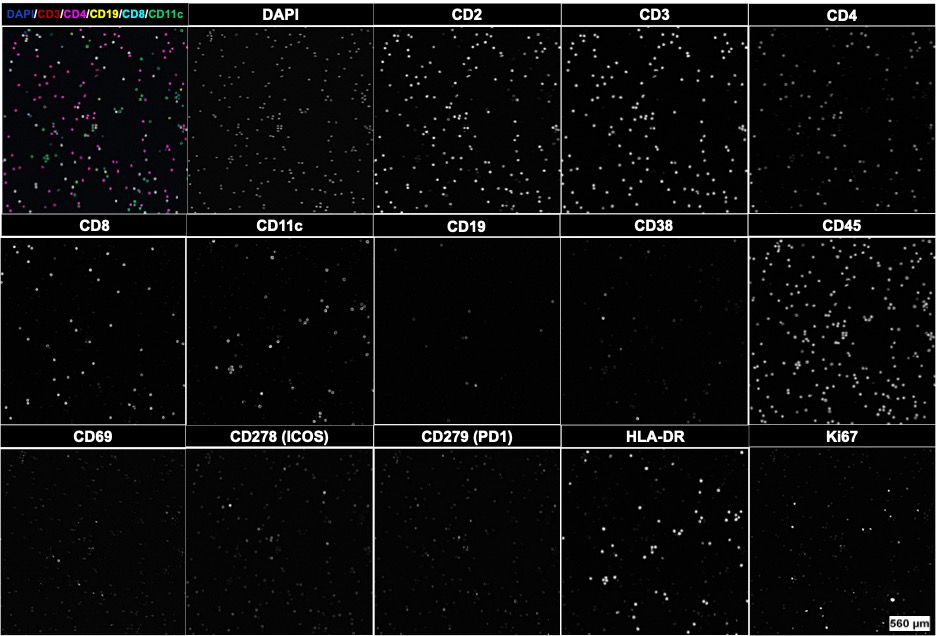


***Figure E1.*** **PhenoCycler representative images.** *(A)* Representative immunoflourescent image of PBMCs stained with 13 antibody panel generated by the PhenoCycler.

***Figure E2.*** **Representation of all 16 subpopulations identified through single cell RNA sequencing across disease.** Uniform manifold plots displaying the contribution of cells from each patient group to the subclusters identified after integrating all 21 single cell RNA samples.


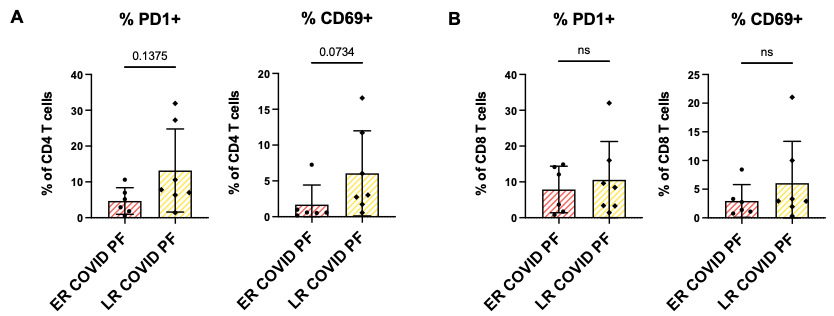


***Figure E3.* T cells in Late Resolving COVID Pulmonary Fibrosis have a trending incraese in activation markers.** Quantification of PD1 and CD69 protein expression of *(A)* CD4 and *(B)* CD8 T cells in Early versus Late Resolving COVID Pulmonary Fibrosis. Mann-Whitney U test was used to test significance. *p≤0.05, ** p≤0.01, and ***p≤0.001. All non-significant values p≤0.15 are shown.

***Figure E4.*** **Dot plot depicting top 20 pathways from gene set enrichment analysis comparing LR COVID PF CD8 T effector cells and non-diseased control.**

***Figure E5.*** **CD8 T cells in Late Resolving COVID Pulmonary Fibrosis have a trending increase in activation markers in comparison to Early Resolving COVID Pulmonary Fibrosis and Idiopathic Pulmonary Fibrosis.** Quantification of *(A)* the percent of HLA-DR+ CD8 T cells within CD3+ T cells, *(B)* percent of HLADR+ CD38+ coexpressing CD8 T cells within CD3+ T cells, *(C)* percent of CD8 T cells co-expressing HLADR+ and CD38+ in Early Resolving COVID Pulmonary Fibrosis (ER COVID PF) versus Late Resolving COVID Pulmonary Fibrosis (LR COVID PF). Quantification of *(D)* the percent of HLA-DR+ CD8 T cells within CD3+ T cells, *(E)* percent of HLADR+ CD38+ coexpressing CD8 T cells within CD3+ T cells, *(F)* percent of CD8 T cells co-expressing HLADR+ and CD38+ in Late Resolving COVID Pulmonary Fibrosis (LR COVID PF) versus Idiopathic Pulmonary Fibrosis (IPF). Mann-Whitney U test was used to test significance. *p≤0.05, ** p≤0.01, and ***p≤0.001. All non-significant values p≤0.15 are shown.

***Figure E6.*** **Decreased expression of HLA-DR in LR COVID PF and IPF are subpopulation specific.** *(A)* Violin plots showing gene expression of MHC-II molecule between control, LR COVID PF, and IPF. Significance for differential expression gene analysis in *A* was determined by non-parametric Wilcoxon rank sum test. Quantification of scRNA-seq relative abundances of monocyte subpopulations *(B)* and total monocytes *(C)* for control, LR COVID PF, and IPF. *B* and *C* significance was tested using Kruskal-Wallis test with Dunn’s multiple comparison test. *p≤0.05, ** p≤0.01, and ***p≤0.001. All non-significant values p≤0.2 are shown.
